# Moral judgment and hormones: A systematic literature review

**DOI:** 10.1371/journal.pone.0265693

**Published:** 2022-04-06

**Authors:** Carolina Coelho Moniz de Campos Freitas, Flávia de Lima Osório

**Affiliations:** 1 Ribeirão Preto Medical School, University of São Paulo, São Paulo, Brazil; 2 National Institute for Science and Technology (INCT-TM, CNPq), Brasília, Brazil; National Institute of Child Health and Human Development (NICHD), NIH, UNITED STATES

## Abstract

This systematic review of the literature aims to evaluate possible associations between moral judgment and hormones. The electronic databases PsycINFO, PubMed, Scielo, Web of Science, Scopus, and LILACS were used. Twenty studies with different methodological designs were reviewed, covering the hormones cortisol, oxytocin, and testosterone, assessing aspects related to polymorphisms in receptor genes, endogenous levels, and exogenous administration. Taken together, the reviewed studies showed a trend towards an association between hormones and moral judgment, with important specificities involving biological, environmental, and individual aspects. Endogenous levels of cortisol, released under stress, showed negative associations with altruistic and utilitarian decisions only in highly emotionally charged dilemmas. Oxytocin receptor gene polymorphisms (rs2268498, rs237889, and rs2254298) and acute administration of this hormone were associated with variability in moral judgment, with sex as an important moderating variable. Testosterone studies have tended to show a positive association with utilitarian moral judgments, particularly in female and in individuals with low prenatal exposure to this hormone. Knowing how hormones influence moral judgment may help expand our understanding of the plurality of human behavior. However, this area of research is new and still little explored, which does not allow for conclusions with a high level of evidence. Subsequent research will benefit from methodological improvements to extend current findings.

## 1. Introduction

Morality can be defined, from an evolutionary perspective, as a set of psychological adaptations that enable cooperation between individuals [1–3], or, more specifically, as a set of customs and values that guide social conduct [4]. Accordingly, moral judgments can be defined as those performed according to moral standards in response to different situations, including the evaluation of a harmful action [5,6], the acceptance of a moral behavior [7–9], or even the position between altruism and egoism in everyday life [10]. Moral judgments differ from other decisions in that moral judgments are associated with concepts such as justice, fairness, and harm [11].

Throughout the history of human thought, various explanations have been given by philosophers such as Plato, Tomas Aquinas, David Hume, Immanuel Kant, John Stuart Mill, Jeremy Bentham, and many others about how moral judgments can or should be arrived [12]. The majority of scientific studies on this topic in the last century followed a rationalistic logic [13]. However, with the advent of neuroscience, several lines of evidence began to attribute an important role to emotions in decision making [14]. Propositions such as the Social Intuitionist Model [15] and the Dual-Process Theory [16], which emphasize a role not only of reason but also of emotions in the process of moral judgment, even at the central level, have gained recognition by highlighting the complexity of this behavior, which is also influenced by a number of personal, biological, and social variables related to the individual who judges and also to the variables of the environment/context in which the action to be judged takes place [6,17–19].

Interest in the influence of these variables on the cognitive and emotional processes associated with moral judgment has grown in recent years, and the results are compelling. For example, men were observed to make more utilitarian judgments than women, which appears to be much more related to the differences in affective responses to harm that have been evidentiated between genders than to cognitive evaluations 4 of consequences [20]. Deontological judgments (more emotionally driven) were more prevalent in women, but only in situations involving the direct infliction of harm, not differing when harm was indirect [21]. Criminal psychopaths judged accidentally committed harm more permissively compared to non-psychopaths, which seemed to be related to the failure of these individuals to assess the emotional aspect of the harm experience of the victim [22]. Also, the combinations of genotypes that enhance dopaminergic signaling selectively increase moral acceptability in females, suggesting that increases in dopamine availability reduce the emotional component of moral decision-making, favoring a more rational decision process [23].

Similarly, the influence of various hormones on moral judgment has also been the focus of some studies, especially in the last two decades. This is possibly due to the association of hormones with a number of other human behaviors, such as fatherhood and motherhood [24,25], sexuality [26], stress [27], affiliation and social cognition [28,29], and others. Studies [30,31] illustrate the influence of testosterone (TES) levels and endogenous cortisol (CORT), respectively, on this process, while Sheele et al. [32] used intranasal administration of oxytocin (OXT).

To the best of our knowledge, the results of studies investigating the associations between hormones and moral judgment have not been systematized to date. Therefore, the aim is to systematically review the literature in this field, taking into account hormone levels, exogenous hormone administration and/or the presence of polymorphisms in hormone receptor genes in order to provide an overview of the scientific production, and highlight the most relevant evidence.

## 2. Method

The methodology of the present study was guided by the Preferred Reporting Items for Systematic Reviews and Meta-Analyses (PRISMA) [33]. The electronic databases PsycINFO, PubMed, Scielo, Web of Science, Scopus, and LILACS were used to search for articles without restriction in terms of language or publication date. The following keywords were used: (moral OR morality) AND (hormone OR oxytocin OR vasopressin OR ("corticotropin releasing") OR ("follicle stimulating") OR ("gonadotropin releasing") OR ("growth hormone") OR luteinizing OR prolactin OR ("thyroid stimulating") OR thyroxine OR ("thyrotropin releasing") OR steroid OR testosterone OR androgen OR estrogen OR progesterone OR glucocorticoid OR cortisol OR angiotensin OR aldosterone). The last search was conducted on January 15, 2021 and the review was registered in PROSPERO (ID: CRD42020193991).

The inclusion criteria were: original articles with observational or experimental design, conducted with adult or adolescent human beings (without sex restriction), whose aim was to evaluate the associations between moral judgment and hormones. The exclusion criteria were: studies that did not use standardized methods for administering exogenous hormones or for measuring concentrations of endogenous hormones.

For hormones, those listed by Norman and Litwack [34] were considered. For the purposes of this study, the terms "moral judgment", "moral evaluation", "moral responsibility", and "moral decision-making" were considered equivalent.

**Fig 1:**
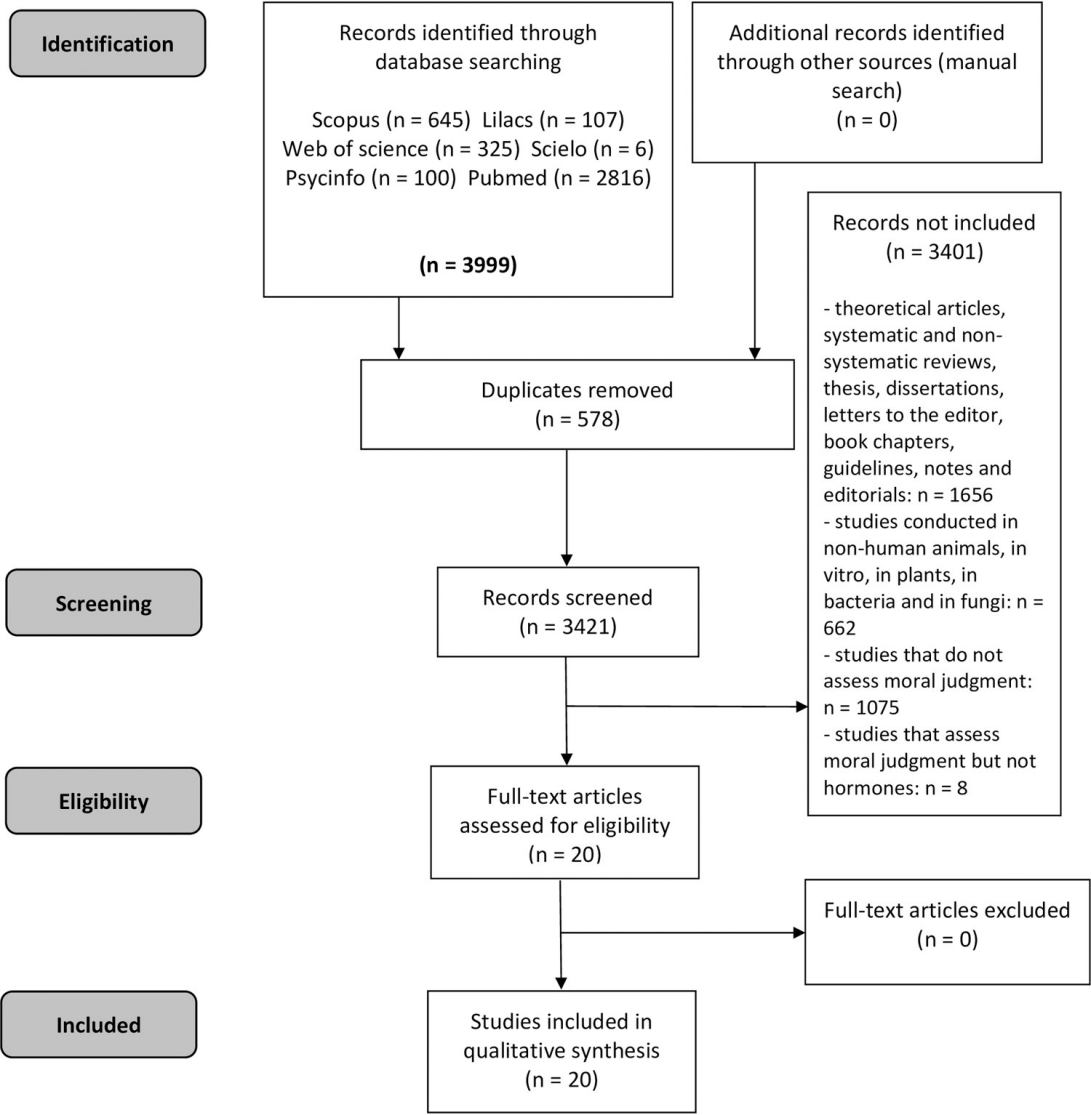
PRISMA flow diagram. Flow diagram illustrating search strategy.

In order to perform data management, the web application Rayyan [35] was used. Two researchers (CCMCCF and FLO) independently decided whether to include articles in the study based on the established criteria, and differences were resolved by consensus. A manual search of the reference lists of the selected articles was also performed as an additional source of data. The detailed process of inclusion and exclusion of studies can be seen in Fig 1.

Data extraction was guided by a standardized table developed by the researchers, focusing on the following variables: (1) authors, year and country of publication of the studies, (2) methodological design, (3) sample characteristics (number of participants, gender/sex, age, education, diagnosis and/or inclusion criteria, source of recruitment, control variables), (4) hormone of interest, methods of analysis of endogenous levels, and/or DNA extraction, and/or form of exogenous administration, and dosage; (5) measurement instrument to assess moral judgment; (6) nature of moral dilemma, (7) main results. The review protocol can be requested from the authors. The analysis of the methodological quality of the studies was also conducted independently by the two researchers using the checklist proposed by National Heart, Lung and Blood Institute [36]. The score was calculated using the mean of the positively scored items, with a higher percentage associated with a higher level of methodological quality.

## 3. Results

A total of 20 articles were analyzed. Six of these focused on CORT, seven on OXT, and seven on TES/androgens. Twelve studies were randomized controlled clinical trials, and eight were observational/cross-sectional studies. The samples were composed entirely of subjects from the general population, mostly university students (n = 16), of both sexes (n = 10), and with a mean age of approximately 30 years (n = 14). Most studies were conducted with subjects from European (n = 11) and North American countries (n = 5). The average sample size was 176 subjects (standard deviation = 211; minimum = 20, maximum = 790). Regarding the types of dilemmas used, sacrificial moral dilemmas predominated (n = 14), but moral responsibility dilemmas (n = 5) and everyday moral dilemmas (n = 4) were also used. More detailed information on sampling and methodological aspects can be found in Table 1 and in S1 File.

**Table 1:** Methodological and sample characteristics of the studies included in this review (N = 20).

Author/Year	Country	Study Design	Interest sample				Comparison sample				Hormone/data collection/analysis or Hormone/route of administration/dose	Moral dilemmas type	MQ
			N/Gender	Age (Years)	Schooling	Diagnostic (Criteria)	N/Gender	Age (Years)	Schooling	Diagnostic (Criteria)			
CORTISOL—ENDOGENOUS HORMONE													
Kossowska et al. (2016) [37]	Poland	CS	70M	23.2 (±2.2)	US	Healthy	-	-	-	-	CORT/Saliva—afternoon/ELISA (DRG Instruments GbH)	SMD	45,45%
CORTISOL—HORONE REACTIVITY													
Starcke et al. (2011) [38]	Germany	RCT-P	11M9F	23.2 (±4.0)	US	Stress Group(induced by TSST); Healthy	11M 9F	24.5 (±3.7)	US	No Stress Group (placebo TSST); Healthy	CORT/Saliva—afternoon/Immunoassay (IBL)—ΔCORT = 33.9%	EMD	28,57%
Youssef et al. (2012) [39]	Trinidad and Tobago	RCT-P	15M 18F	18–27	UGS	Stress Group (induced by TSST); Healthy	15M 17F	18–27	UGS	No Stress Group (placebo TSST); Healthy	CORT/Saliva—morning/Immunoassay (Salimetrics)—ΔCORT = 63.2%	SMD	28,57%
Singer et al. (2017) [31]	Germany	RCT-P	30M	18–28	US	Stress Group (induced by TSST); Healthy	20M	18–28	US	No Stress Group (placebo TSST); Healthy	CORT/Saliva—afternoon/ TRFIA (DELFIA)—ΔCORT = 68.5%	EMD	28,57%
Singer et al. (2020) [40]	Germany	RCT-P	20M	18–37	US	Stress Group (induced by TSST); Healthy	20M	18–37	US	No Stress Group (placebo TSST); Healthy	CORT/Saliva—afternoon/ TRFIA (DELFIA)—ΔCORT = 118.8%	EMD	35,71%
Singer et al. (2021) [41]	Germany	S1-RCT-C	50M 49F	18–35	NI	Stress Group (induced by TSST)/ No Stress Group (placebo TSST); Healthy	-	-	-	-	CORT/Saliva—afternoon/ TRFIA (DELFIA)—ΔCORT = 108,5%	EMD	42,85%
		S2- RCT-C	40M 40F	18–35	NI	Stress Group (induced by TSST)/ No Stress Group (placebo TSST); Healthy	-	-	-	-			
OXYTOCIN—RECEPTOR GENE													
Walter et al. (2012) [42]	Germany	CS	28M 122F 4 NI	21.9 (±4.5)	US	Healthy	-	-	-	-	OXTR rs2268498/ Buccal swabs/ PCR (Light Cycler System 1.5—Roche Diagnostics)	MRD	40,00%
Bernhard et al. (2016) [44]	USA	CS	S1: 159F, 115M	24.2 (NI)	14.9y	Healthy	-	-	-	-	OXTR 25 SNPs/NI/PCR (Sequenom Iplex)	SMD	40,00%
		CS	S2: 211F 159M	26.3 (NI)	15.5y	Healthy	-	-	-	-	OXTR rs237889/NI/PCR (Sequenom Iplex)	SMD	
Shang et al. (2017) [43]	China	CS	256M 534F	16.5 (±0.7)	HS	Healthy	-	-	-	-	OXTR rs2254298, rs2268498/ Buccal cells/ PCR (FlexiGene DNA Kit)	MRD	40,00%
Palumbo et al. (2020) [45]	Italy	CS	129M	52,0 (±9.1)	13.9y	Insurance brokers Healthy (MCMI-III)	109M	48.6 (±7.7)	12.0y	Other professions; Healthy (MCMI-III)	OXTR rs53576, rs2268498, rs:1042770/ Saliva/ PCR (DNA Genotek)	SMD	54,54%
OXYTOCIN—EXOGENOUS HORMONE													
Preckel et al. (2014) [46]	Germany	RCT-P	25M	25.0 (±4.7)	NI	Healthy	23M	24.1 (± 4.5)	NI	Healthy	OXT/Nasal spray/ 24IU (SD) PLA^1^	SMD	35,71%
Scheele et al. (2014) [32]	Germany	RCT-P	37M 30F	24.4 (±3.0) 23.2 (±2.9)	16.6y 16.4y	Healthy (DSM-IV) (SCID-I/ SCID-II)	37M 30F	25.2 (±2.6) 23.73 (±2.8)	17.1y 16.3y	Healthy (DSM-IV) (SCID-I/ SCID-II)	OXT/Nasal spray/ 24IU (SD) PLA^1^	SMD	28,57%
Goodyear et al. (2015) [47]	USA	RCT-P	42M	18–30	US	Healthy	42M	18–30	US	Healthy	OXT/Nasal spray/40IU (SD) PLA^3^	MRD	35,71%
TESTOSTERONE—RECEPTOR GENE													
Gong et al. (2017) [48]	China	CS	111M 328F	20.4 (± 1.2)	US	Healthy (Symptom Check List 90)	-	-	-	-	AR CAG/ Hair follicule cells/ PCR (Chelex-100)	SMD	40,00%
TESTOSTERONE—ENDOGENOUS HORMONE													
Carney and Mason. (2010) [30]	USA	CS	85M 32F	28.0 (NI)	GS	NI	-	-	-	-	TES/Saliva/ ELISA (Salimetrics)	SMD	36,36%
Chen et al. (2016) [49]	Taiwan	RCT-C	20F	20–30	NI	Healthy	-	-	-	-	TES/Saliva/ELISA (IBL)	SMD	35,71%
Arnocky et al. (2017) [51]	Canada	RCT-C	30M	18–35	US	Healthy	-	-	-	-	TES/Blood/ ELISA (DRG International)	SMD	35,71%
Brannon et al. (2019) [52]	USA	RCT-P	58M 42F	19.3 (±1.9)	US	NI	58M 42F	19.3 (±1.9)	US	NI	TES/ Saliva/ ELISA (DRG)	SMD	78,57%
Armbruster et al. (2021) [50]	Germany	CS	71M 86F	25.3 (± 4.3) 23.31 (± 3.4)	US	Healthy	-	-	-	-	TES/Saliva/SaliCaps (IBL)	SMD	45,45%
TESTOSTERONE—EXOGENOUS HORMONE													
Montoya et al. (2013) [53]	Netherlands	RCT-C	20F	18–30	US	Healthy	-	-	-	-	TES /Sublingual/ 0.5mg (SD) PLA^2^	SMD	42,85%
Chen et al. (2016) [49]	Taiwan	RCT-C	20F	20–30	NI	Healthy	-	-	-	-	TES/ Sublingual/ 0.5 mg (SD) PLA^2^	SMD	35,71%
Arnocky et al. (2017) [51]	Canada	RCT-C	30M	18–35	US	Healthy	-	-	-	-	TES/Gel/150mg (SD) PLA^3^	SMD	35,71%
Brannon et al. (2019) [52]	USA	RCT-P	58M 42F	19.3 (±1.9)	US	NI	58M 42F	19.3 (±1.9)	US	NI	TES/ Nasal spray /14mg (SD) PLA^2^	SMD	78,57%

*CAG = an androgen receptor gene polymorphism; CORT = cortisol;* CS = cross-sectional study; CNI = consequences, norms and inaction; DNA = Deoxyribonucleic acid; EIA = enzyme immunoassay; ELISA = enzyme-linked immunosorbent assay; EMD = everyday moral dilemmas; F = females; M = males; IU = international *unit; MCMI-III = Millon Clinical Multiaxial Inventory-III*; MQ = methodological quality; *MRD = moral responsibility dilemmas;* NI = not informed; OXT = oxytocin; *OXTR = oxytocin receptor gene;* PCR = *polymerase chain reaction*, PLA = placebo; *RCT-C = randomized controlled trial with cross-over design; RCT-P = randomized controlled trial with parallel design; S = study; SD = single dose;* SMD = sacrificial moral dilemmas; SNP = single nucleotide polymorphisms; TES = testosterone; *TRFIA =* time-resolved fluorescent immunoassay; *TSST = Trial Social Stress Test*; UGS = undergraduate student; *US = university student; y = years;* 1 = sodium chloride solution; 2 = vehicle; 3 = NI; ΔCORT = variation of cortisol from basal level to the beginning of the moral judgment task, after TSST or placebo.

The mean quality score of the cross-sectional studies was 43%. The main critical issues were the absence of information on dropout rates and blinding of outcome assessors, the absence of sample size justification and/or power description, and the use of instruments without prior psychometric studies to assess outcomes. Randomized controlled trials had a mean methodological quality score of 38%. The same limitations described for the group of studies above were observed in addition to the lack of information on the randomization methods used, allocation concealment, dropouts and adherence to the study protocol, and the lack of intention-to-treat analysis method. For detailed information, see S2 File.

Regarding the outcomes of interest, only studies involving the association of moral judgment with one of these three hormones were found: CORT, OXT, and TES, the results of which are presented separately, see Tables 2–4.

**Table 2:** Main results of the studies on cortisol included in this review.

Author/ Year	Aim	Main Results	*r*	*effect size*	*p-value*
ENDOGENOUS HORMONE					
Kossowska et al. (2016) [37]	To examine whether the effects of individual variation in stress levels, measured by CORT level, on moral decisions depended on individual differences (need for closure)	• Need for closure mediates the relationship between CORT and moral decisions:			
		• CORT was linked to utilitarian decisions at high need for closure level (only to ingroup dilemmas)			.02*
		• CORT was linked to deontological decisions at low need for closure level (only to no-ingroup dilemmas)			.02*
HORMONE REACTIVITY					
Starcke et al. (2011) [38]	To examine whether stress affects moral decision-making	• Stressed group: ↑ CORT level while performing the task: ↓ altruistic decisions only in high-emotional moral dilemmas	-.56		< .05*
		• No-stress group: No significative correlations between CORT level and decisions in high/low emotional moral dilemmas	-.13/ -.01		.59/ -96
Youssef et al. (2012) [39]	To evaluate if stress could influence moral decision-making	• Stressed group: ↓ utilitarian choices as compared to the control group in personal moral dilemmas (differences remain for separate analyzes between the gender)			.02*
		• AUC CORT response was correlated with utilitarian responses to personal moral dilemmas	-.27		.03*
		• likelihood to make utilitarian decisions: male group> female group			< .01*
Singer et al. (2017) [31]	To investigate the impact of acute stress on everyday moral decision-making	Stressed group: ↑ CORT level while performing the task: ↑ altruistic decisions	.35		.01*
		• Mean CORT level while performing the task explained 7% of the observed variance of the percentage of altruistic decisions (independent predictor)	ΔR^2^ = .07		.03*
		• CORT level was not correlated with decision certainty and feelings	-.08/ .07		> .58
Singer et al. (2020) [40]	To access the relation between everyday moral decision-making and acute psychosocial stress and how it is influenced by effects of social closeness	• Stressed group: ↑ CORT level while performing the task		ds ≥ .83	≤ .01*
		• CORT level: no significative association with moral decision-making (socially close/distant protagonist)	≤ 0.23		≥ .15
Singer et al. (2021) [41]	To evaluate the association between moral decision-making and gender, personality and CORT after stress exposure or placebo	• Study 1			
		• CORT: higher in the stress than control condition			> .05
		• Correlation between CORT and moral decision-making was nonsignificant	rs ≤ ∣.16∣		≥ .13
		• No gender-specific correlations between CORT and moral decision-making	rs ≤ ∣.10∣		≥ .48
		• Agreeableness had a significant impact on moral decision-making only in the stress condition	β = .20		.04*
		• Study 2			
		• Stress group: female group: ↑ CORT AUCg: ↑ altruism	.34		.04*

AUC—area under the curve; AUC_G_—areas under the curve with respect to ground; CORT = cortisol; d = Cohen’s d; r = Pearson’s r; ΔR2 = delta R-squared; β = beta; % = percentage; ↑ = increase of; ↓ = decrease of

* = statistical significance.

**Table 3:** Main results of the studies on oxytocin included in this review.

Author/Year	Aim	Main Results	*r*	*effect size*	*p-value*
RECEPTOR GENE					
Walter et al. (2012) [42]	To evaluate the associations between the polymorphism rs2268498 on OXTR gene and moral judgment	• **rs2268498** Blameworthiness for accidentally committed harm: CC/CT > TT		ƞ2 = .07	.001*
		Blameworthiness for intended and committed harm: CC/CT = TT		ƞ2 < .001	> .05
		Blameworthiness for intended but failed harm: CC/CT = TT		ƞ2 = .003	> .05
Bernhard et al. (2016) [44]	Study 1: To evaluate the associations between 25 polymorphisms on OXTR gene and moral judgment	Study 1 • **rs237889**: utilitarian responses: CC > TT (this effect persisted when including sex, age or mood as covariates)		β = -.16	< .02*
		• No associations between moral judgments and the other OXTR SNPs: rs237877, rs6777088, rs13093809, rs7629329, rs17049505, rs1042778, rs237888, rs4686301, rs2268491, rs2268492, rs2268494, rs11131149, rs53576, rs2268495, rs237897, rs237899, rs237902, rs4686302, rs4643699, rs401015, rs237922, rs2270465, rs6443206, rs237924			> .05
	Study 2: To replicate Study 1 to evaluate the associations between moral judgment and rs237889 polymorphism on OXTR gene	Study 2 • **rs237889**: utilitarian responses: CC > TT (original set of high-conflict dilemmas + medical dilemmas)		β = -.15	.007*
		• No influence of age or mood			< .02*
		• Males utilitarian responses > females			< .01*
Shang et al. (2017) [43]	To evaluate the association between the OXTR gene polymorphisms rs2254298 and rs2268498, and prosociality mediated by moral evaluation.	•**rs2268498**: Prosocial judgment of moral questions: CT> CC		d = .24	.04*
		No interaction between moral evaluation, genotype and gender		ƞ2p = .004	.24
		• **rs2254298:** Prosocial judgment of moral questions: only males: G_ > AA		d > .48	< .002*
Palumbo et al. (2020) [45]	To investigate whether OXTR polymorphisms (rs53576, rs2268498, rs1042770) are associated to insurance brokers moral judgment	• **rs53576:** Maximizing harm choices: Insurance brokers: GG = A_ / Other professions: GG = A_			> .05
		Moral acceptability: Insurance brokers: GG_ = A_ / Other professions: GG = A_			> .05
		• **rs2268498:** Maximizing harm choices: Insurance brokers: C_ = TT/ Other professions: C_ = TT			> .05
		Moral acceptability: Insurance brokers: C_ = TT/ Other professions: C_ = TT			> .05
		• **rs1042778:** Maximizing harm choices: Insurance brokers: GG = A_/ Other professions: GG = T_			> .05
		Moral acceptability: Insurance brokers: GG = T_/Other professions: GG = T			> .05
		• OXTR score profiles**: • Maximizing harm choices: Insurance Brokers: low = high /Other professions: low = high			> .05
		Moral acceptability: Insurance Brokers: low < high / Other professions: low = high			.02*/ > .05
EXOGENOUS HORMONE					
Preckel et al. (2014) [46]	To investigate the modulatory effects of OXT on the emotional ambivalence by using moral dilemmas	• OXT x PLA: No significant effect on deontological/ utilitarian response rate		d = .09	.77
		• OXT group: ↓ Neural response to ambivalent moral dilemmas in anterior/ medial/ posterior cingulate cortex, precuneus and orbitofrontal cortex			
		• PLA group: No difference in speed of acceptance of moral dilemmas (utilitarian/ deontological responses)			.73
		• OXT group: Accepted moral dilemmas (utilitarian response) significantly faster than rejected them (deontological response)		d = .11	.04*
Scheele et al. (2014) [32]	To investigate whether OXT influences self-referential processing in moral decision making in male and female participants	• Male group: OXT: ↑ approval of self-benefit items only in personal moral dilemmas		d = .58	.02*
		• Male group: OXT did not enhance the reaction time differences for self-benefit dilemmas compared to non-self-benefit			.16
		• Female group: OXT: ↓ approval of self-benefit items only in personal moral dilemmas		d = .65	.02*
		• Female group: OXT: ↑ reaction time for self-benefit dilemmas compared to non-self-benefit		d = .82	.02*
Goodyear et al. (2015) [47]	To investigate the effects of intranasal OXT on intuitions about the relationship between free will and moral responsibility	• PLA: Responsibility ratings for offenses in the indeterministic universe group > deterministic universe group		d = 1.0	.003*
		• OXT: Responsibility ratings for offences in the indeterministic universe group = deterministic universe group		d = .10	.77
		• Moral responsibility ratings in the indeterministic universe: OXT < PLA (about 15%)		d = .70	.04*
		• Moral responsibility ratings in the deterministic universe group: OXT = PLA		d = .30	.27

d = Cohen’s d; OXT = oxytocin; OXTR = oxytocin receptor gene; PLA = placebo; r = Pearson’s r; β = beta; ƞ2 = eta squared; ƞ2p = partial eta squared; % = percentage; ↑ = increase of; ↓ = decrease of

* = statistical significance

** = based on the functional effect of each variation of OXTR signaling or based on the literature data concerning genetic associations with moral behavior, empathy and prosocial behavior.

**Table 4:** Main results of the studies on testosterone included in this review.

Author/Year	Aim	Main Results	*r*	*effect size*	*p-value*
RECEPTOR GENE					
Gong et al. (2017) [48]	To investigate whether CAG polymorphism in androgen receptor gene is associated with moral judgment	• Moral dilemma task: • Female group: permissibility in utilitarian moral decisions: SS > LL / SL = SS / SL = LL		d = .33	.015*/ .44/ .11
		• Male group: permissibility rating in utilitarian moral decisions: S = L			.77
		• Moral transgression task: • Female group: Genotype S_: higher permissibility of accidentally committed harm but not to attempted but failed harm/ intentionally committed harm	R^2^ = .02		.008*/ .54/ .83
		• Male group: Genotype S: not a predictor of permissibility ratings of accidentally committed harm/ attempted but failed harm/ intentionally committed harm			> .11
ENDOGENOUS HORMONE					
Carney and Mason (2010) [30]	To evaluate the association between moral judgment and endogenous TES levels	• TES levels: Intransigent utilitarians > fair-weather utilitarians. This effect was greater for the female group compared the male group.	.18, .30/ .18		< .05* < .11
		Individuals who endorsed pushing the man in the footbridge dilemma > individuals who endorsed not pushing the men	.19		< .05*
		No relation between response to switch dilemma and TES (all sample)			> .60
Chen et al. (2016) [49]	To examine the neuromodulatory effect of testosterone in young females by combining moral dilemmas, 2D: 4D, functional magnetic resonance imaging (fMRI), and subjective ratings of morally laden scenarios (secondary data)	• TES levels: positively related to impersonal moral permissibility judgments, but not to personal moral permissibility judgments (inevitable or evitable harm)	.52/ < .12		.02*/ >.65
Arnocky et al. (2017) [51]	To investigate the effects of TES administration and endogenous TES on moral judgments, and whether these effects are mediated by prenatal sex-hormone priming in male (secondary data)	• TES level: marginally and negatively associated with utilitarian responses			.08
Brannon et al. (2019) [52]	To investigate the effects of TES administration and endogenous TES on moral judgments (secondary data)	• Sensitivity to moral norms: TES high level < TES low level		d = .44	.003*
		• Sensitivity to consequences and preference for inaction: TES high level = TES low level		d = .03/ .17	.82/ .24
		• Preference for action judgements on moral dilemmas in which a proscriptive norm prohibits action and the benefits of action outweigh its costs to well-being: TES high level = TES low level		d = 0.26	.09
Armbruster et al. (2021) [50]	To investigated moral judgments in men, free menstrual cycling women and contraceptive users, and whether these correlations are mediated by endogenous testosterone.	• Free menstrual cycling women: • Positive correlation between TES level and utilitarianism		r = .30	.05*
		• No significant correlation between TES and deontology			.74
		• Contraceptive users: No significant correlation between TES and utilitarianism or deontology.			≥.30
		• Male group: negative correlation between TES and deontology. No significant correlation between TES and utilitarianism		r = -.23	.05*/ .53
EXOGENOUS HORMONE					
Montoya et al. (2012) [53]	To investigate the effects of TES administration on moral judgments on female subjects, and whether these effects are mediated by prenatal sex-hormone priming (2D:4D)	• Moral permissibility judgments: TES = PLA (any dilemma category)		ƞ2p = .001	.90
		• Dilema Type vs. TES-PLA vs. 2D:4D: • Impersonal dilemmas: no main effects		ƞ2p = .00	1.00
		• Personal dilemmas involving evitable harm: no main effects		ƞ2p = .00	.93
		• Personal dilemmas involving inevitable harm 2D:4D predicts an increase in moral permissibility following TES relative to • PLA:		ƞ2p = .45/ r = 0.67	.001*
		• Subjects showing an increase in utilitarian judgment after TES have 2D:4D greater than the mean			.04*
		• Subjects showing a decrease in utilitarian judgment after TES have 2D:4D marginally significantly lower than the mean			.06
Chen et al. (2016) [49]	To examine the neuromodulatory effect of testosterone in young females by combining moral dilemmas, 2D: 4D, functional magnetic resonance imaging (fMRI), and subjective ratings of morally laden scenarios	• Utilitarian judgments on personal evitable harm dilemmas: TES > PLA			.002*
		• Utilitarian judgments on non-moral/ impersonal/ personal-Inevitable harm dilemmas: TES = PLA			>.09
		• Dilema Type vs. TES-PLA vs. 2D:4D:			
		• 2D:4D positively explained 22% of the variance in the effect of TES administration on the utilitarian judgments of personal-evitable dilemmas	.47		.05*
		• 2D:4D negatively explained 27% of the variance in the effect of TES administration on the utilitarian judgments of impersonal dilemmas	-.52		.03*
		• 2D:4D negatively explained 23% of the variance in the effect of TES administration on the utilitarian judgments of non-moral dilemmas	-.48		.05*
		• 2D:4D: no correlation in the effect of TES administration on utilitarian judgment in personal-inevitable dilemmas	.03		.92
		• High 2D:4D group:—TES tended to reduce impersonal permissibility judgements			.08
		• —TES: higher punishment			
		• TES administration: • Intentional harm: ↑ activity in the amygdala, anterior insular cortex, and dorsolateral prefrontal cortex, vmPFC			
		• Accidental harm: ↓ activity in the amygdala, anterior insular cortex, and dorsolateral prefrontal cortex, vmPFC			
Arnocky et al. (2017) [51]	To investigate the effects of TES administration and endogenous TES on moral judgments, and whether these effects are mediated by prenatal sex-hormone priming in male	• Utilitarian response for Incidental others/ self, instrumental others/ self dilemmas: TES = PLA			1.00
		• Dilema Type vs. TES-PL vs. 2D:4D: 2D:4D ratio did not interact with drug condition to predict moral decision making and did not predict variability in moral decision making			> .46
Brannon et al. (2019) [52]	To investigate the effects of TES administration and endogenous TES on moral judgments	• Dilema Response vs. TES-PLA • preference for action judgements on dilemmas in which a proscriptive norm prohibits action and the benefits of action outweigh its costs to well-being (traditional analysis: utilitarianism): TES < PLA		d = .37	.009*
		• Sensitivity to moral norms: TES > PLA		d = .46	.001*
		• Sensitivity to consequences: TES = PLA		d = .26	.07
		• Preference for inaction: TES = PLA		d = .02	.88

d = Cohen’s d; PLA = placebo; r = Pearson’s r; R2 = R-squared; TES = testosterone; 2D:4D = second-to-forth digit ratio; ƞ2p = partial eta squared; % = percentage; ↑ = increase of; ↓ = decrease of

* = statistical significance.

### 3.1. Cortisol and moral judgment

This group of studies consisted of six articles, and only one of them [37] directly assessed associations between baseline CORT levels and moral judgment through an observational design. The other studies (experimental) aimed to evaluate the effects of acute stress on moral judgment, with CORT being the secondary outcome, as it is considered one of the biomarkers of this condition (stress reactivity). CORT was assessed in all studies in this category (n = 6) by saliva using immunoassay methods. The mean variation from basal CORT levels to CORT levels after stress induction was 78,58% (SD = 32.81).

In two mixed-sample studies, results showed that the increase in CORT levels under stress was weakly/moderately associated with a decrease in altruistic [38] and utilitarian [39] judgments only in the face of dilemmas with specific characteristics (everyday moral dilemmas/high emotional dilemmas and sacrificial moral dilemmas/personal dilemmas, respectively). In three other studies, led by the same group of researchers, and using everyday moral dilemmas [31,40,41], the results had a different direction, as an increase in altruistic responses was observed under stress, which was not always associated with CORT levels. The first association found was expressive for the male sample (r = 0.35) [31], whereas, in the second study [41], it was only meaningful for the female sample (r = 0.34). In the third study [40], there were no significant correlations in an exclusively male sample. The results of both groups were not associated with variations in CORT levels.

The findings of the only study to assess basal levels of CORT (male sample) [37], the findings were controversial and dependent on personal subject characteristics, in this case, the need for closure. For subjects with high levels of this trait (expressed by a high need for certainty regarding decisions), CORT levels were associated with utilitarian decisions in the face of ingroup dilemmas, whereas for subjects with low need for closure, CORT levels were associated with deontological decisions in the face of no-ingroup dilemmas.

### 3.2. Oxytocin and moral judgment

The studies on OXT and moral judgment involved polymorphisms of the OXT receptor gene (n = 4) and exogenous administration of this hormone (n = 3). As in the genetic studies, different types of sample materials were collected and then analyzed by the polymerase chain reaction method. A total of 27 single nucleotide polymorphisms (SNP) were evaluated in samples with different characteristics. In a study conducted in adult Caucasian participants [42], both genders C-allele carriers (SNP rs2268498) rated accidentally committed harm as more blameworthy than non-carriers. There were no differences between C-allele carriers and carriers of the TT- genotype regarding intentional harm or failed attempts to cause harm. In contrast, in the study conducted with Chinese adolescents [43], only carriers of the CT-genotype in this SNP judged moral dilemmas more prosocially. In an Italian study by [44], no association was found between OXT receptor gene polymorphisms and utilitarianism, either in a specific sample of male insurance brokers or in male subjects from the general population.

The OXT receptor gene rs2254298 polymorphism was examined in the study by Shang et al. [43], in which male carriers of the G-allele judged moral issues more prosocially. As for the rs237889 polymorphism, in the study by Bernhard et al. [44], carriers of the CC-genotype made more utilitarian judgments compared to carriers of the TT-genotype regardless of sex, but only in high conflict dilemmas. No difference was found for other polymorphisms studied (n = 25) [44,45].

Studies that resorted to the administration of exogenous OXT (N = 3) used the intranasal route and a single dose of 24IU or 40IU. The administration of OXT (24IU) did not alter moral judgment (utilitarian vs. deontological) [46], but decreased the activation of neural regions associated with ambivalence (anterior, posterior, and medial cingulate cortex; precuneus; and orbitofrontal cortex) in an exclusively male sample [46]. After administration of OXT (24IU), there was an increase in self-benefit responses to moral dilemmas in males but a decrease in females [32]. Finally, after administration of OXT (40IU), participants considered the offender to be more morally responsible when acting in an indeterministic context (i.e., with free will), which was not the case in deterministic contexts [47].

### 3.3. Testosterone/Androgens and moral judgment

The studies in this group include the assessments of an androgen receptor gene polymorphism (CGA; n = 1), endogenous TES levels (n = 5), and exogenous administration of TES (n = 4).

Regarding the androgen receptor gene CAG polymorphism, the genetic material extracted from the hair follicle cells was analyzed by polymerase chain reaction technique [48]. The results showed that, only for female, the SS-genotype (related to a greater availability of TES) was associated with more utilitarian judgments, especially in accidentally committed harm scenarios [48].

Studies evaluating endogenous TES predominantly used saliva samples and the enzyme-linked immunosorbent assay technique to measure hormone levels. The results showed a trend of association between high TES levels and utilitarian responses [30,49,50], but with specificities regarding the type of dilemma (only personal dilemmas in Carney and Mason [30], and only impersonal dilemmas in Chen et al. [49]) and gender (in Carney and Mason [30] the results were more expressive for women and in Armbruster et al. [50] the results were only significant for women who did not use oral contraceptives). For men, the results were not significant in either Armbruster et al. [50] or Arnocky et al. [51] studies. In the study by Brannon et al. [52] (mixed sample) subjects with higher TES levels showed lower sensitivity to norms.

Among studies using exogenous TES, there is diversity in the routes of administration used and dosages, as well as in the results. In two [49,53], conducted with women only, sublingual administration of 0.5mg TES was associated with an increase in utilitarian responses to personal dilemmas, depending on dilemma type (evitable in Chen et al. [49] and inevitable in Montoya et al. [53]). In both studies, the influence of 2D:4D ratio on utilitarian judgments was observed with larger effects for subjects with high 2D:4D (lower prenatal TES exposure).

It was also found that, after TES administration, activity in the amygdala, anterior insular cortex, dorsolateral prefrontal cortex, and ventromedial prefrontal cortex was increased in situations of intentional harm and decreased in situations of accidental harm [49].

In a study conducted with males only [51], administration of 150 mg of TES in gel to the skin was not associated with a significant effect on moral judgment even after accounting for prenatal exposure to TES. In other study [52], which involved the administration of 14 mg of TES via nasal spray to a mixed gender sample, results suggest effects of TES in the increase of inaction in dilemmas where proscriptive norm prohibits action and the benefits of action outweigh its costs to well-being. The same study also showed that sensitivity to norms was greater in the group of subjects who received TES.

## 4. Discussion

The findings of this review suggest that the hormones studied tend to influence moral judgments, as they do in many other human behaviors [54–60].

Studies on CORT and moral judgment have focused primarily on stress reactivity, as acute stress conditions lead to activation of the sympathetic nervous system and release of CORT through the activation of the HPA axis [61,62]. However, the results shown have been specific and controversial. This is because, in two studies [38,39], an increase in CORT levels was associated with a decrease in altruistic and utilitarian decisions in highly emotionally charged dilemmas, in other words, it disfavored decisions that focused on well-being. These findings can be supported by the Dual Process Theory [7,16], which postulates the action of two neural systems in moral judgment: a rational system (involving awareness and rational evaluation of facts, which tends to favor utilitarian decisions) and an emotional system (based on affective responses, especially when the individual is emotionally involved in the situation, leading to more deontological responses). Stressful situations favor the operation of automatic/intuitive affective responses, as they evoke emotions, especially of a negative nature, such as fear, which activate different brain areas of the limbic system [63,64] and interfere with the rational/reflective decision-making process, leading to more deontological and egoistic responses, to the detriment of more utilitarian and altruistic responses [38,39].

In contrast, the results of Singer et al. [31,40,41] were more inconsistent and, when significant, demonstrated an increase in altruistic decisions associated with an increase in CORT levels [31,41]. Nevertheless, these authors share the arguments described above regarding the moral judgment process, with the difference that, for them, and in line with the "Stress Induced Deliberation-to-Intuition" model [65], in the face of aversive/stressful situations, regular and automatic affective responses are paramount, favoring innate behavioral responses that have been empirically demonstrated to be predominantly prosocial at this level [66].

Moreover, they emphasize that the controversies between the findings are related to the moderating role of different variables such as the type of dilemma and the social proximity to the characters involved (e.g., participants decided more altruistically in scenarios involving socially close protagonists [40]), time to decision/judgment after stress [41], gender [20], and individual behavioral traits (e.g., high levels of empathy, agreeableness, and social desirability may favor altruistic responses) [67–69]. These observations are consistent with the study in which only baseline CORT levels were measured [37] and in which the results were explicitly dependent on personality and on the context/character of the dilemma. These findings point to the complexity of factors involved in the moral decision-making process, whether at the biological, environmental or personal level [70], and call attention to the need for better control of these variables in future studies.

It is important to note that the effects of CORT on decision-making have been reported previously in the evaluation of patients with Cushing’s syndrome (who have higher basal CORT levels) [71]. They showed impairment in this function as their decisions were driven by short-term reward and long-term punishment and may improve with treatment [71,72].

Regarding OXT, this hormone is known to influence a wide repertoire of social behaviors such as trust, cooperation, perspective taking, and empathy [48,55,73,74], which may affect the process of moral judgment. The studies analyzed here have shown that OXT receptor gene polymorphisms (rs2268498, rs237889, and rs2254298) may be associated with variability in moral judgment, reinforcing the role of heritability in this behavior [75] and the prosocial role of OXT, although results are still incipient. Previous studies have pointed to the influence of OXT receptor gene polymorphisms on other human behaviors and traits, such as sexual behavior [76], empathy [48,64,77,78], emotional face recognition [79], and prosociality [80].

Again, it is worth highlighting the importance of contextual variables and individual subject characteristics, which appear to modulate outcomes related to OXT in this context as well, as previously pointed out by Bartz et al. [81]. For example, in the results related to OXT administration, a lower attribution of responsibility was favored in indeterministic scenarios, which stimulated motivational affiliation [47]. In the face of personal moral dilemmas, the use of exogenous OXT showed opposite effects depending on sex, which may indicate sex-specific evolutionary mechanisms, as males were more likely to make selfish decisions, possibly in an attempt to defend their offspring, whereas females were more likely to make altruistic decisions, possibly to promote caring and survival [32]. The indirect effects of OXT at the neural level suggest a reduction in ambivalence in the face of conflict [46], which would alleviate emotional distress, as already pointed out in other studies, e.g., on trust [55].

On the other hand, androgens are also highly correlated with different aspects of human behavior, especially those associated to moral judgment, such as empathy, processing of emotional stimuli, stress, and risk aversion [29,50,82,83]. In the studies analyzed here, there was a tendency for an association between TES and utilitarian decisions, whether at endogenous, exogenous or genetic levels, despite the specificities associated with the type of dilemma (i.e., context) and, above all, with sex, as these associations are more pronounced in female. For authors such as Carney and Mason [30] and Gong et al. [48], the reason for this is that TES reduces sensitivity to affective signals (especially the negative ones, such as the harm done to someone) that would stimulate empathic behaviors and decisions (focused on victims), thus favoring decisions that are less affective and more focused on outcomes, which increases the instrumentalization of decisions. It has also been postulated that TES may decrease activity in the ventromedial prefrontal cortex [30], which could also contribute to more utilitarian responses [14], as sensitivity to important social and somatic signals would be decreased [14,84]. Recent findings [49] extend this hypothesis, as a decrease in activity in neural circuits related to moral evaluation (amygdala, anterior insular cortex, dorsolateral prefrontal cortex, ventromedial prefrontal cortex) and in the connectivity between amygdala with the rostral dorsolateral and dorsomedial prefrontal cortexes was found in the face of accidentally committed harm (when harm was intentional, the findings were opposite), confirming the previous findings [85] that the specific effects of TES on amygdala activation are mediated by motivation. In the cited study, TES administration decreased amygdala activity during threat avoidance, whereas activation was observed in threat approach situations [85].

It is worth highlighting that the effects of TES on moral judgment were different when prenatal exposure to this hormone was taken into account, as the 2D:4D ratio explained some of the variance in the data associated with the effect of TES administration [49,50], reinforcing previously observed findings concerning, for example, fear [86] and affective empathy [87], suggesting neurodevelopmental effects of prenatal exposure to androgens in adulthood at both neural and behavioral levels [49,88,89]. TES more markedly increases moral permissiveness in high 2D:4D individuals (low prenatal exposure to TES), possibly by reducing fear and affective empathy. These findings also support the results highlighted here that higher endogenous TES levels are associated with greater utilitarianism [30,49,50].

Regarding the influence of the gender variable, the fact that the results for female samples were more expressive seems to be related to the lower availability of this hormone in women compared to men [90] and also to the existing relationship between moral judgment and gender [20,21,70,91]. Women are more inclined to reject harm and action in moral dilemmas, thus showing a deontological bias, whereas men have a more utilitarian bias [20,91]; this difference is more pronounced when the dilemmas have high emotional salience [21].

Considering that moral judgments are complex processes influenced by a number of variables at individual, environmental, social, cultural and biological levels [92–95], this study sought to highlight the influence of hormones on this process, which may help to expand the understanding of the plurality of human behavior (see S3 File). This area of study is new and still under-researched, which does not allow conclusions to be drawn with a high level of evidence. The results, which are still in the early stages, indicate the existence of direct/indirect associations between the hormones studied and judgment regarding situations of a moral nature. Attention is drawn to the very limited number of hormones studied, the use of different methodologies, significant methodological weaknesses, and the predominant use of populations from European and North American countries, which should be overcome in future studies designed to replicate and extend the current findings and providing more specific evidence of the possible influence of cultural aspects on this process. In addition, studies involving the interaction of different factors such as biological, cultural, social, individual, and environmental variables are also advisable to assess the complexity of this area of study.

## Supporting information

S1 Checklist (DOCX)

S1 File Other methodological and sample characteristics of the studies included. (DOCX)

S2 File Methodological quality. (DOCX)

S3 File Graphical abstract. (PDF)
