# Supplementary material for: Moral judgment and hormones: A systematic literature review
Source: PLoS One. 2022 Apr 6;17(4):e0265693. doi: 10.1371/journal.pone.0265693 (PMC8985980; doi:10.1371/journal.pone.0265693)
Supplement: S1 File — (DOCX) [file pone.0265693.s002.docx]

| **Supplementary Material - Other methodological and sample characteristics of the studies included in this review** | | | | | |
| --- | --- | --- | --- | --- | --- |
| **Author/**  **Year** | **Control Variables** | **Recruitment**  **(Interest samples/ Comparison sample)** | **Tools** | | |
|  |  |  | Name/source | **Type** | **Specificities** |
| CORTISOL - ENDOGENOUS HORMONE | | | |  |  |
| Kossowska et al. (2016) | Gender (only men), need for closure | UN | from Greene et al. (2001) | SMD | 4 dilemmas, ingroup/ no-ingroup |
| CORTISOL – HORMONE REACTIVITY | | | |  |  |
| Starcke et al. (2011) | menstrual cycle | UN/UN | Everyday Moral Decision-Making Task (EMDM; C. Polzer, personal communication) | EMD | 20 dilemmas, highly/ low emotional |
| Youssef et al. (2012) | Gender | UN/UN | based on Greene et al. (2007) | SMD | 30 dilemmas, moral/ non-moral, personal/ impersonal |
| Singer et al. (2017) | Gender (only men) | UN/UN | based on Sommer et al. (2010) | EMD | 28 dilemmas |
| Singer et al. (2020) | Gender (only men); ethnicity (only Germans), social closeness | UN/UN | Everyday Moral Conflict Situation Scale (EMCS Scale; Singer et al. 2019) | EMD | 20 dilemmas, socially close/ distant protagonist |
| Singer et al. (2021) | S1/S2 - Gender, menstrual cycle, personality | NI | Everyday Moral Conflict Situation Scale (EMCS Scale; Singer et al. 2019) | EMD | 20 dilemmas, socially close/ distant protagonist |
| OXITOCYN - RECEPTOR GENE | | | |  |  |
| Walter et al. (2012) | Race (only white) | UN | based on Young & Saxe (2009) | MRD | 24 dilemmas, all-neutral/ accidental harm/ attempted harm/ intentional harm |
| Bernhard et al. (2016) | S1 - Gender; race (only white), age, mood | COM | from Greene et al. (2001, 2004, 2008) | SMD | 36 dilemmas, personal/ impersonal, high/ low-conflict |
|  | S2 - Gender, race (only white), age, mood | NI | from Ransohoff (2011) | SMD | 12 dilemmas, medical |
| Shang et al. (2017) | Gender, ethnicity (only Chinese), kinship (unrelated) | HS | based on Keller et al. (2003) | MRD | moral transgression/ moral choice |
| Palumbo et al. (2020) | Gender (only men), race (only white), ethnicity (Italian), kinship (unrelated) | NI/NI | from Lotto, Manfrinati & Sarlo (2014) | SMD | 24 dilemmas, instrumental/ incidental |
| OXYTOCIN - EXOGENOUS HORMONE | | | |  |  |
| Preckel et al. (2014) | Gender (only men) | NI/NI | from Harrison et al. (2012). Greene et al. (2001) | SMD | 48 dilemmas, moral/ non-moral |
| Scheele et al. (2014) | Gender; menstrual cycle | UN/UN | from Greene et al. (2001 | SMD | 62 dilemmas, moral/ non-moral, personal/ impersonal, self-benefit/ non-self-benefit |
| Goodyear et al. (2015) | - | UN/UN | from Robinson & Kurzban, 2007 | MRD | 10 dilemmas, criminal offences, high harm/ low harm, deterministic/ indeterministic universes |
| TESTOSTERONE - RECEPTOR GENE | | | |  |  |
| Gong et al. (2017) | Gender, ethnicity (Chinese Ham) | UN | from Greene et al. (2001, 2004) | SMD | 16 dilemmas, personal/ impersonal |
|  |  |  | based on Young & Saxe (2009) | MRD | 24 dilemmas, all-neutral/ accidental harm/ attempted harm/ intentional harm |
| TESTOSTERONE - ENDOGENOUS HORMONE | | | |  |  |
| Carney & Mason. (2010) | Gender | UN | 2 dilemmas^14^, SMD, personal/ impersonal |  |  |
| Chen et al. (2016) | Gender (only women), 2D:4D digit ratio | NI | from Greene et al. (2009) | SMD | 48 dilemmas, non-moral/ impersonal, personal-inevitable/ personal-evitable, balanced on emotional intensity |
|  |  |  | from Decety et al. (2012), Yoder & Decety (2014a), Yoder & Decety (2014b) | MRD | 96 dilemmas, intentional/ unintentional, people/objects |
| Arnocky et al. (2017) | Gender (only men), 2D:4D digit ratio | UN | from Lotto, Manfrinati & Sarlo (2014) | SMD | 16 dilemmas, incidental others/ self, instrumental others/ self |
| Brannon et al. (2019) | Gender | UN | CNI Model dilemmas (from Gawronski et al., 2017) | SMD | 24 dilemmas, proscriptive norm prohibits action/ prescriptive norm prescribes action x benefits of action greater than costs/ benefits of action smaller than costs |
| Armbruter et al. (2021) | Gender, menstrual cycle | UN | from Conway & Gawronski (2013) | SMD | 20 dilemmas, congruent/ incongruent |
| TESTOSTERONE - EXOGENOUS HORMONE | | | |  |  |
| Montoya et al. (2012) | Gender (only women), 2D:4D digit ratio | UN | based on Greene et al. (2001, 2004) | SMD | 12 dilemmas, personal/ impersonal, evitable/ inevitable, balanced on emotional intensity |
| Chen et al. (2016) | Gender (only women), 2D:4D digit ratio | NI | from Greene et al. (2009) | SMD | 48 dilemmas, non-moral/ impersonal, personal-inevitable/ personal-evitable, balanced on emotional intensity |
|  |  |  | from Decety et al. (2012), Yoder & Decety (2014a), Yoder & Decety (2014b) | MRD | 96 dilemmas, intentional/ unintentional, people/objects |
| Arnocky et al. (2017) | Gender (only men), 2D:4D digit ratio | UN | from Lotto, Manfrinati & Sarlo (2014) | SMD | 16 dilemmas, incidental others/ self, instrumental others/ self |
| Brannon et al. (2019) | Gender | UN | CNI Model dilemmas (from Gawronski et al., 2017) | SMD | 24 dilemmas, proscriptive norm prohibits action/ prescriptive norm prescribes action x benefits of action greater than costs/ benefits of action smaller than costs |
| BMI = body mass index; COM = community; HS = high school students; CNI = consequences, norms and inaction; EMD = everyday moral dilemmas; MRD = moral responsibility dilemmas; NI = not informed; SMD = sacrificial moral dilemmas; S1: study one; S2: study two; TSST = Trial Social Stress Test; UN = university; 2D:4D: 2 to 4 digit ratio | | | | | |
