# Supplementary material for: Moral judgment and hormones: A systematic literature review
Source: PLoS One. 2022 Apr 6;17(4):e0265693. doi: 10.1371/journal.pone.0265693 (PMC8985980; doi:10.1371/journal.pone.0265693)
Supplement: S2 File — (DOCX) [file pone.0265693.s003.docx]

| **Supplementary Material B - Methodological quality assessment adopting the National Heart, Lung and Blood Institute checklists** | | | | | | | | | | | | | | | | | |  |
| --- | --- | --- | --- | --- | --- | --- | --- | --- | --- | --- | --- | --- | --- | --- | --- | --- | --- | --- |
| **Hormone** | **Author/Year** | **Study Design** | **1** | **2** | **3** | **4** | **5** | **6** | **7** | **8** | **9** | **10** | **11** | **12** | **13** | **14** | **Score** | **Quality Rating*** |
| **CORT** | Kossowska et al. (2016) | CS | YES | YES | NR | NA | NO | NO | NO | NA | YES | NR | YES | NR | NA | YES | 45,45% | fair |
|  | Starcke et al.  (2010) | RCT-P | YES | NR | NR | NO | NR | YES | NR | NR | NR | YES | NO | NO | YES | NR | 28,57% | poor |
|  | Youssef et al.  (2011) | RCT-P | YES | NR | NR | NO | NR | YES | NR | NR | NR | YES | NO | NO | YES | NR | 28,57% | poor |
|  | Singer et al.  (2017) | RCT-P | YES | NR | NR | NO | NR | YES | NR | NR | NR | YES | NO | NO | YES | NR | 28,57% | poor |
|  | Singer et al.  (2020) | RCT-P | YES | NR | NR | NO | NR | YES | NR | NR | NR | YES | YES | NO | YES | NR | 35,71% | poor |
|  | Singer et al.  (2021) | RCT-C | YES | NR | NR | NO | NR | YES | NR | NR | NR | YES | YES | YES | YES | NR | 42,85% | fair |
| **OXT** | Walter et al.  (2012) | CS | YES | YES | NR | NA | NO | NO | NO | NA | YES | NA | NO | NR | NA | YES | 40,00% | poor |
|  | Bernhard et al.  (2016) | CS | YES | YES | NR | NA | NO | NO | NO | NA | YES | NA | NO | NR | NA | YES | 40,00% | poor |
|  | Shang et al.  (2017) | CS | YES | YES | NR | NA | NO | NO | NO | NA | YES | NA | NO | NR | NA | YES | 40,00% | poor |
|  | Palumbo et al.  (2020) | CS | YES | YES | NR | YES | NO | NO | NO | NA | YES | NA | YES | NR | NA | YES | 54,54% | fair |
|  | Preckel et al.  (2014) | RCT-P | YES | NR | NR | YES | NR | YES | NR | NR | NR | YES | NO | NO | YES | NR | 35,71% | poor |
|  | Scheele et al.  (2014) | RCT-P | YES | NR | NR | YES | NR | YES | NR | NR | NR | YES | NO | NO | NO | NR | 28,57% | poor |
|  | Goodyear et al.  (2015) | RCT-P | YES | NR | NR | YES | NR | YES | NR | NR | NR | YES | NO | NO | YES | NR | 35,71% | poor |
| **TES** | Gong et al.  (2017) | CS | YES | YES | NR | NA | NO | NO | NO | NA | YES | NA | NO | NR | NA | YES | 40,00% | poor |
|  | Carney & Mason. (2010) | CS | YES | YES | NR | NA | NO | NO | NO | NA | YES | NO | NO | NR | NA | YES | 36,36% | poor |
|  | Armbrust et al.  (2021) | CS | YES | YES | NR | NA | YES | NO | NO | NA | YES | YES | NO | NR | NA | YES | 45,45% | fair |
|  | Chen et al.  (2016) | RCT-C | YES | NR | NR | YES | NR | YES | NR | NR | NR | YES | NO | NO | YES | NR | 35,71% | poor |
|  | Arnocky et al.  (2017) | RCT-C | YES | NR | NR | YES | NR | YES | NR | NR | NR | YES | NO | NO | YES | NR | 35,71% | poor |
|  | Brannon et al.  (2017) | RCT-P | YES | YES | YES | YES | NR | YES | YES | YES | YES | YES | NO | YES | YES | NR | 78,57% | good |
|  | Montoya et al.  (2012) | RCT-C | YES | NR | YES | YES | NR | YES | NR | NR | NR | YES | NO | NO | YES | NR | 42,85% | fair |
| CS = cross-sectional study; NA = not applicable; NR = not reported; RCT-C = randomized controlled trial crossover; RCT-P = randomized controlled trial parallel; 1-CS = Was the research question or objective in this paper clearly stated?; 1-RCT = Was the study described as randomized, a randomized trial, a randomized clinical trial, or an RCT?; 2-CS = Was the study population clearly specified and defined?; 2-RCT = Was the method of randomization adequate (i.e., use of randomly generated assignment)?; 3-CS = Was the participation rate of eligible persons at least 50%?; 3-RCT = Was the treatment allocation concealed (so that assignments could not be predicted)?; 4-CS = Were all the subjects selected or recruited from the same or similar populations (including the same time period)? Were inclusion and exclusion criteria for being in the study prespecified and applied uniformly to all participants?; 4-RCT = Were study participants and providers blinded to treatment group assignment?; 5-CS = Was a sample size justification, power description, or variance and effect estimates provided?; 5-RCT = Were the people assessing the outcomes blinded to the participants' group assignments?; 6-CS = For the analyses in this paper, were the exposure(s) of interest measured prior to the outcome(s) being measured?; 6-RCT = Were the groups similar at baseline on important characteristics that could affect outcomes (e.g., demographics, risk factors, co-morbid conditions)?; 7-CS = Was the timeframe sufficient so that one could reasonably expect to see an association between exposure and outcome if it existed?; 7-RCT = Was the overall drop-out rate from the study at endpoint 20% or lower of the number allocated to treatment?; 8-CS = For exposures that can vary in amount or level, did the study examine different levels of the exposure as related to the outcome (e.g., categories of exposure, or exposure measured as continuous variable)?; 8-RCT = Was the differential drop-out rate (between treatment groups) at endpoint 15 percentage points or lower?; 9-CS = Were the exposure measures (independent variables) clearly defined, valid, reliable, and implemented consistently across all study participants?; 9-RCT = Was there high adherence to the intervention protocols for each treatment group?; 10-CS = Was the exposure(s) assessed more than once over time?; 10-RCT = Were other interventions avoided or similar in the groups (e.g., similar background treatments)?; 11-CS = Were the outcome measures (dependent variables) clearly defined, valid, reliable, and implemented consistently across all study participants?; 11-RCT = Were outcomes assessed using valid and reliable measures, implemented consistently across all study participants?; 12-CS = Were the outcome assessors blinded to the exposure status of participants?; 12-RCT = Did the authors report that the sample size was sufficiently large to be able to detect a difference in the main outcome between groups with at least 80% power?; 13-CS = Was loss to follow-up after baseline 20% or less?; 13-RCT = Were outcomes reported or subgroups analyzed prespecified (i.e., identified before analyses were conducted)?; 14-CS = Were key potential confounding variables measured and adjusted statistically for their impact on the relationship between exposure(s) and outcome(s)?; 14-RCT = Were all randomized participants analyzed in the group to which they were originally assigned, i.e., did they use an intention-to-treat analyses?; * = studies scoring less than 40% were considered “poor”, 41% to 70% “fair”, and 71% to 100% “good”. | | | | | | | | | | | | | | | | | | |
