# Supplementary figures and images for: Moral judgment and hormones: A systematic literature review
Source: PLoS One. 2022 Apr 6;17(4):e0265693. doi: 10.1371/journal.pone.0265693 (PMC8985980; doi:10.1371/journal.pone.0265693)

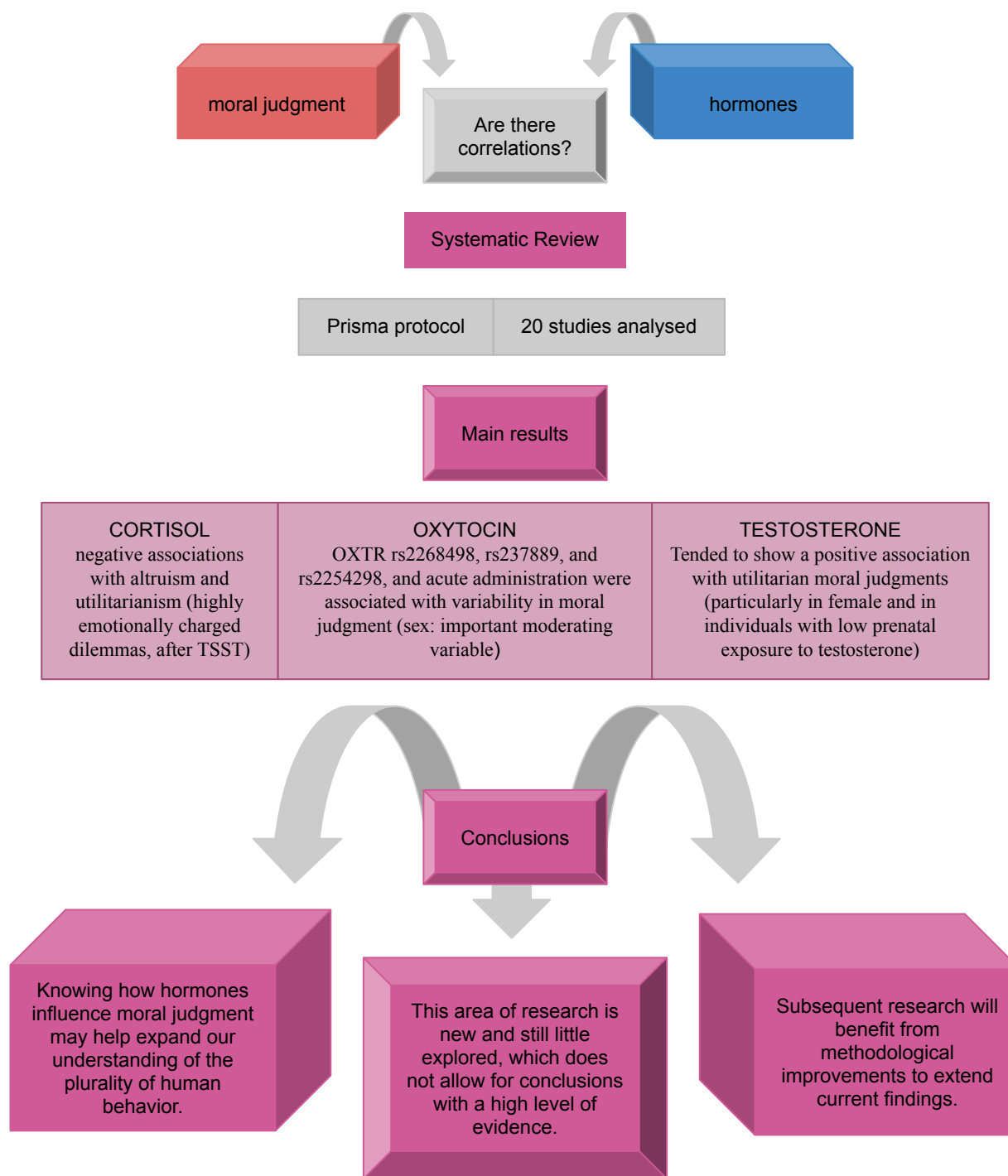

Supplement: S3 File — (PDF) [file pone.0265693.s004.pdf]
